# The Influence of the Surface Topographical Cues of Biomaterials on Nerve Cells in Peripheral Nerve Regeneration: A Review

**DOI:** 10.1155/2021/8124444

**Published:** 2021-07-24

**Authors:** Fang Liu, Jiawei Xu, Linliang Wu, Tiantian Zheng, Qi Han, Yunyun Liang, Liling Zhang, Guicai Li, Yumin Yang

**Affiliations:** ^1^Key Laboratory of Neuroregeneration of Jiangsu and Ministry of Education, Nantong University, 226001 Nantong, China; ^2^Coinnovation Center of Neuroregeneration, Nantong University, 226001 Nantong, China; ^3^School of Medicine, Nantong University, 226001 Nantong, China; ^4^NMPA Key Laboratory for Research and Evaluation of Tissue Engineering Technology Products, Nantong University, 226001 Nantong, China

## Abstract

The surface topographies of artificial implants including surface roughness, surface groove size and orientation, and surface pore size and distribution have a great influence on the adhesion, migration, proliferation, and differentiation of nerve cells in the nerve regeneration process. Optimizing the surface topographies of biomaterials can be a key strategy for achieving excellent cell performance in various applications such as nerve tissue engineering. In this review, we offer a comprehensive summary of the surface topographies of nerve implants and their effects on nerve cell behavior. This review also emphasizes the latest work progress of the layered structure of the natural extracellular matrix that can be imitated by the material surface topology. Finally, the future development of surface topographies on nerve regeneration was prospectively remarked.

## 1. Introduction

Peripheral nerve injury is the most common disease in clinical medicine, which brings much trouble to the patients in their life. The pathogenic factors are varied, including trauma, traffic accidents, or artificially excised nerve tissue by surgery, sports injury, etc. [1]. Although most of the peripheral nerve damage will not endanger a human's life, it could hinder the function of normal surrounding tissues [2], lead to mobility difficulties and sensory abnormalities, and have a negative impact on people's daily life. From a biological point of view, nerve damage and scarring at the site of injury are major contributors to the healing of damaged nerves, which may limit the functional recovery of peripheral nerves [3]. Therefore, repairing peripheral nerves remains an urgent clinical problem that has not been adequately addressed. In today's medical field, the functional repair of damaged tissues in the nervous system is still a huge problem. After nerve tissue damage, the formed glial scar will produce inhibitory molecules to block the regeneration of nerve axons [4]. In order to achieve the functional replacement of damaged tissues in neural tissue, more and more artificial nerve grafts made of synthetic or natural biomaterials have been developed to promote the growth, proliferation, and differentiation of nerve cells [5] .

Tissue engineering is aimed at solving the problem of organ shortage by developing biological substitutes that can help restore or enhance the function of damaged tissue [6]. With the continuous deepening of the research on tissue engineering, the research on nerve grafts is also transitioning from simple primary preparation to advanced bionic and functional preparation [7]. More and more studies have shown that the microenvironment constructed by the surface properties of tissue engineering product materials has an important impact on tissue engineering and organ regeneration [8–10]. For example, biomaterials not only provide mechanical and three-dimensional structural support for tissue and organ regeneration, but some of its own properties and factors can also regulate tissue and organ regeneration. These characteristics and factors include the surface characteristics of materials (such as surface topology), which are now gradually becoming a research hotspot [8–10]. The surface topology of a material plays a very important role in guiding cell behavior, including morphology, adhesion, differentiation, and axon guidance [8, 11]. Nerve cells or neurons are responsible for transmitting signals to other cells. They collect signals through dendrites and cell soma and transmit signals through synapses at the end of axons [12]. Therefore, numerous studies have been devoted to the manufacture of the surface pattern of biomaterials for exploring the role of contact guidance. In recent years, two-photon polymerization nanolithography micro/nanomanufacturing technology has become a powerful and useful manufacturing tool, which can generate two-dimensional (2D) to three-dimensional (3D) arbitrary micro/nanotopologies of various materials with high spatial resolution, thus arousing great interest in cell and tissue engineering [13].

Material surface topology was reported to have a significant effect on nerve regeneration [6, 14]. The normal nerve tissue is distributed on a long-strip structure with good orientation growth. Thus, simulating this structure will be beneficial for nerve regeneration. Miller et al. found that the stripe-shaped distribution of the PDLA pattern could regulate the orientation growth behavior of Schwann cells, which had the best alignment when the groove width was 10-20 *μ*m, but the depth of the grooves showed little effect [15]. Song and Uhrich studied the size effect of laminin micropatterns on the axon growth rate, length, and tropism of DRG; they found that when the protein pattern width was 40 *μ*m, DRG cells had the fastest axon growth rate and best orientation [16]. The above two studies indicated that when the appropriate topography size, i.e., approaching to cell size, was designed, the process of nerve regeneration could be significantly promoted. The studies have an important reference value for revealing the effect of the surface size of the biomaterial on nerve regeneration. Blong et al. found that the patterned surface of collagen and laminin could better regulate the orientation and distribution of neural progenitor cells but had little effect on their differentiation ability [17]. Researchers have also used micrographic methods to successfully differentiate neural stem cells into neural cells, astrocytes, and oligodendrocytes [18]. The latest report by Liu et al. displayed that the 3D topology of biomaterials (porosity, pore size, etc.) and the geometric topology of subcell size could affect the specific differentiation of progenitor cells and stem cells [19]. In addition, Lu et al. found that basal stiffness and topology can coordinately regulate stem cell morphology and differentiation [20]. Therefore, the micropatterns on the surface of biomaterials can build a physical microenvironment that is conducive to cell growth and orientation, to promote and regulate the growth and spatial distribution of nerve cells for achieving better and faster nerve regeneration. However, numerous studies are currently limited to the regulation of topographies on cell morphology, while the molecular mechanism of how nerve cells sense the surface topology of materials, and how surface topology affect the inner reaction of cells, such as gene variation and signal pathway activation, is still not fully understood.

**Figure 1:**
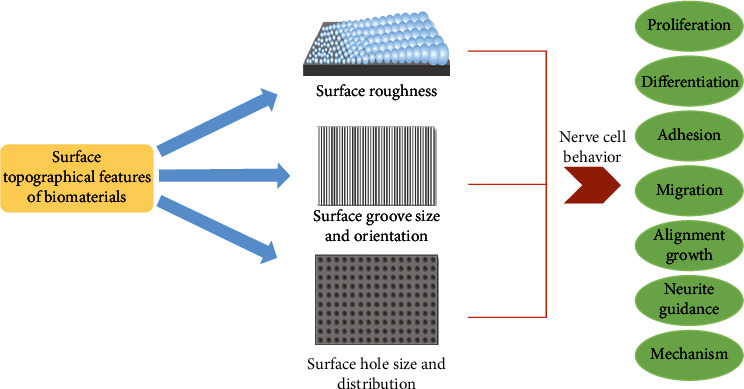
Schematic diagram of the influence of the surface topographical features of biomaterials on the behavior of nerve cells.

Thus, understanding how nerve cells respond to different biomaterial surface topologies is critical to the development of suitable nerve grafts used in regenerative medicine and tissue engineering. The purpose of this review is mainly to emphasize the significance of the surface topology of biomaterial implants and their effect on the behavior of nerve cells, including proliferation, differentiation, adhesion, migration, alignment growth, and neurite guidance and relevant mechanism (Figure 1).

## 2. Different Features of Topographies and Their Influence on Cell Behavior

### 2.1. Topography Affects Cell Proliferation and Differentiation

Cell proliferation is one of the important physiological functions of living cells and an important life characteristic of organisms, which is the basis of organism growth, development, reproduction, and heredity, and could be seriously affected by the structure of the surrounding microenvironment. In recent years, many studies have applied various microfabrication methods to prepare concrete topographical maps, at sub-micro- and microscales. A new technology has been developed to prepare microgrooves with adsorbed proteins on a biodegradable polymer substrate made of poly(D,L-lactic acid) [6, 10, 15, 21]. The effect of matrix-mediated chemical and physical guidance on the growth and arrangement of Schwann cells in vitro was studied. The preparation of surface nanotopography by Onesto et al. and others can guide nerve cells to assemble into efficient computational networks, providing new tools and standards for tissue engineering and regenerative medicine [6, 10, 15, 21]. Generally, the natural physiological tissues have a specific microtopological structure, with the repetition or regular arrangement of certain geometric features in space. The morphology and structure are closely related to the physiological functions it undertakes. Besides, the specific microtopology could provide space for cell growth and diffusion in the tissue. The difference of microtopology in different tissues may be one of the factors that regulate the biological behavior of cells. As a substrate for promoting cell or tissue growth, microtopological structures have begun to be used in various tissue engineering fields. According to the natural structure of physiological tissues, various types or sizes of porous or microgrooved arrays for regulating cell or tissue growth have been designed and fabricated [22, 23]. Previous studies have shown that microtopology could regulate the growth of nerve cells and tissues and promote cell proliferation and differentiation. The suitable topographical types fabricated on biomaterials can be used to regulate cell expansion, direction differentiation, and migration [24, 25]. Moreover, understanding the mechanism of cell surface topographical interaction is of great significance for scaffold design in nerve tissue engineering [26, 27].

Many studies have shown that microtopography can promote the proliferation of adherent cells including most nerve cells. In previous studies, a simple method of combining freeze-drying and micromolding to efficiently and expandably manufacture a biomaterial conduit with a seamless sidewall and longitudinally arranged structures on the inner wall was developed. The highly arranged microstructures could speed up the directional growth of neonatal nerve tissues, and the foraminous sidewalls were expected to be in favor of loading biological factors and reducing nutrient leakiness or axon outgrowth [28]. The number of proliferating cells was largely dependent on the diameter of the nanofibers, and the proliferation increases with the decrease of the fiber size. Christopherson et al. also found a similar phenomenon by culturing rat neural stem cells on a grid composed of nanofibers, which further confirmed the extreme sensitivity of cell proliferation to specific sizes of nanoscale features [29].

In numerous differentiation clues such as ECM composition, soluble factors, material component, and structure, the nanomorphology around the cell plays a vital role [30]. Embryonic stem cells (ESC), as a renewable source of cells, have great application potential in nerve injury repair. In order to study the response of ESC to uneven surfaces, Chen et al. [31] used a simple self-assembly method to prepare highly ordered hexagons with completely opposite curvatures. The array and honeycomb structure surface were formed by the cell sequence, and experiments proved that in the concave honeycomb structure, the cells could not enter the honeycomb pores, but penetrate the honeycomb pores, which promoted the elongation of cells. The release of gravity promoted homosexual diffusion and cell proliferation. Alvarez et al. [32] designed a biomimetic PLA nanofiber scaffold that could release L-lactic acid. The topological structure of PLA nanofibers could promote the growth of neurons and glial cells. They showed that the release of L-lactic acid through the bionic scaffold composed of electrospun PLA fibers reproduced the three-dimensional organization and supported functions of embryonic radial glial cells, thereby simulating physical and biochemical characteristics of the embryonic NSC niche. Although there is still a long way before clinical transformation, the results of the research opened unexpected and exciting prospects for the design of cell-free implant devices. By promoting glial cell generation, neurogenesis, and vascularization, the functional nerve tissue lost after injury can be restored without growth factors, genetic manipulation, or exogenous cells.

Cheong et al. [33] designed natural polymer scaffolds with bionic topologies. They found that a mussel adhesion protein could be fused with biological functional peptides in the extracellular matrix to strengthen differentiation and proliferation of neuron and Schwann cells. Additionally, the contact between nerve cells and aligned nanofibers can greatly promote functional recovery after nerve regeneration. Therefore, by synergistically providing physical adhesion for cell proliferation, integrin-mediated stimulation for cell differentiation, and contact guidance for cell alignment, the proposed multidimensional bioinspiration strategy realized by aligned nanofiber scaffolds can be an effective and universal bioinspired method in the field of neuroregenerative medicine. However, because the secretion of mussel protein is very low, the price of products extracted directly from mussels is relatively expensive, and it is thus very complicated to obtain genetically engineered products with the characteristics of natural adhesion proteins [34]. Islam et al. [35] conducted the first neuron-like PC12 growth study on untreated and easily available micro-nano-surfaces. Microreactive ion etching provided a fast and economical method to fabricate a nanostructured substrate for neuron-like cell culture. It was found that even in the absence of any extracellular matrix treatment (such as collagen and laminin), nanostructures could stimulate the growth of cultured PC12 cells. The neuron-like structure on the nanostructured glass cover slip was increased by 200% compared with the ordinary glass cover slip. Morphological studies showed that the attachment and growth of PC12 cells on the nanostructured substrate did not initiate any apoptosis mechanism of the cells. Coupled with the ability to induce and enhance the proliferation of PC12 cells, these substrates had excellent potential for neural cell attachment. Neural cell attachment is a precursor for enhanced differentiation, which can be used to manipulate axon regeneration and guide neural circuit reconstruction, thus showing a great potential for treatment of nervous system trauma. Nanostructures can also promote the differentiation of mouse embryonic stem cells into neuronal lineages. For example, the diameter of the nanofibers affected the differentiation of neural progenitor cells in rats. The differentiation of oligodendrocytes on 283 nanofibers was increased by 40%, and differentiation of neurons on 749 nanofibers was increased by 20%, in contrast to the standard tissue culture surface [29].

The topographic signal of the substrate will have a certain guiding role in the process of cell development and tissue repair, and control the fate of cells. Moe et al. [36] fabricated a simple, customizable, cost-effective topology array that can combine different nano- to microtopologies with various aspect ratios and complexity levels. Anisotropic topographic maps (2-micron gratings, nanometer gratings) and isotropic topographic maps (1-micron column) promoted neuron differentiation. The isotropic 1-micron column and 2-micron holes were conducive to the differentiation of primary glial cells. In general, it showed that a multiarchitecture chip (MARC) could identify the best combination of topographical and biochemical cues by analyzing different topographies at the same time, which is beneficial for the cell-topography interaction. Therefore, the customizable MARC as a topological chip was a novel and unique platform with a wide range of potential biological applications. Choi et al. [37] used the engineered concave microarray to regulate the formation of the shape and size of the intermediate products of embryonic stem cells. The PDMS was used as the base material, and the differentiation of cardiomyocytes and neurons was induced by controlling the size of the concave microarray, which proved that the size of the concave microarray may be a main factor in regulating the differentiation of embryonic stem cells and a potential tool to guide the fate of embryonic stem cells. Fernandez et al. [38] designed and produced polycaprolactone/polyethylene oxide–polycaprolactone-blended fiber materials using electrospinning technology, and it was found that this blend promoted the growth of rat neural stem cells by culturing neural stem cells isolated from mouse brain fiber materials, and neural stem cells even differentiated into neurons and astrocytes without adding any growth factors. Tachizawa et al. [39] used hydroxyl propyl cellulose (HPC) and sodium alginate (Na-Alg) to prepare a neuronal cell-oriented scaffold with a bundle-like structure. These bundled fibers allowed neuronal cells to follow the axon direction and fiber long axis elongation, and experiments had shown that human-induced pluripotent stem cells could successfully differentiate into neuronal cells based on the fiber bundle structure. In addition, microtopology can effectively adjust to the differentiation and proliferation of cells [40]. In clinic, on the basis of effective treatment, microtopology could provide a way to enhance the differentiation and proliferation of nerve cells in order to reduce the damage of nerve tissue defects to the human body [41, 42]. The foundation of the topographical structure shortens the treatment time and improves the treatment efficiency. A list of the impacts of the topographical structure on the proliferation and differentiation of nerve cells is presented in Table 1.

### 2.2. Topography Affects Cell Adhesion and Migration

Adhesion is an important condition for anchoring dependent cell survival, growth, and proliferation on the matrix. Poor adhesion may lead to cell immobility or even apoptosis [43]. Cell adhesion is the basis of communication between cells and the external environment and plays a major role in organizational development [44]. Thus, the adhesion is considered to be the first indicator of the interaction of cells with their surrounding environment, prior to other properties, such as proliferation, differentiation, and migration. Early studies showed that the surface topographical properties of biomaterials could guide nerve cell adhesion and the migration process [45]. The topographic maps submitted through nanofibers had been shown to promote the regeneration of peripheral nerves with a damaged gap. However, its basic mechanism has not yet been fully elucidated. For example, topography [9], wettability [46], chemical composition [47] are usually interrelated properties. An increase in the hydrophobicity of the surface of a fluorinated polystyrene material could cause a decrease in human MG63 osteosarcoma cell adhesion [46]. Adsorption of fibronectin (FN) on the hydroxyl functional groups (functional group as compared with -CH_3_) showed high levels of *α*_5_*β*_1_ level, resulting in an increase of cell adhesion and the structural strength of the signal component levels associated with focal adhesion of cells [48]. The topographies have a huge influence on cell adhesion and migration behavior. Jia et al. [49] prepared neatly arranged poly(L-lactic acid-co-e-caprolactone) (P(LLA-CL)) nanofibers. The Schwann cells that migrated and the number of axons on the neatly arranged nanofibers were 2.0 times and 2.84 times that of the random group, respectively. The nerve guide channels constructed by the aligned nanofibers obviously enhance peripheral nerve regeneration through stimulating a more promacrophage phenotype.

Radhakrishnan et al. [50] used polylactide-glycolide copolymers to prepare random and longitudinally arranged electrospun fibers including two-dimensional films and three-dimensional scaffolds. Compared with two-dimensional films and random fibers, oriented nanofiber scaffolds showed a significant increase in the adhesion of Schwann cells after culture for 3, 6, and 12 hours, respectively. The orientation structure of the scaffold promoted the maturation of Schwann cells, thereby promoting the formation of the myelin sheath to maintain Schwann cells' function. A new method for preparing polycaprolactone scaffolds that mimic the natural structure of peripheral nerves with a micropatterned ridge/groove structure on the surface by combining electrospinning and micromolding has previously been developed. The micropatterned polycaprolactone scaffold exhibits a porous structure, and its hydrophobicity and mechanical properties increased with the electrospinning flow rate of the polycaprolactone solution, while the chemical properties did not change. The micropatterned polycaprolactone scaffold had good stability and could effectively regulate the attachment and orientation of Schwann cells in the early stage after cell culture. The results showed that the flow rate of the PCL electrospinning solution had an important influence on the morphology of the scaffold and the adsorption behavior of cells and proteins. The micropatterned scaffold with a flow rate of 0.12 mL/h^−1^ had a good regulation effect on the adhesion and arrangement of Schwann cells, without negatively affecting the normal biological functions of the cells. This research could provide important design concepts for artificial nerve implants, and provide an experimental and theoretical foundation for the development of implants in tissue engineering [51]. Although polycaprolactone has excellent mechanical properties and plasticity, PCL's biological properties and functions are low, and the degradation rate in the body is slow. Thus, the performance of PCL including biocompatibility and biodegradation should be further improved for future applications [52]. Xie et al. [53] used electrospinning technology to prepare a single layer of nonoriented and neatly arranged nanofibers for dorsal root ganglion (DRG) culture, and it was proved that on nonoriented nanofibers, the DRG neurites spread radially from the body with no particular orientation. In contrast, when DRG was cultured in single-layer-oriented nanofibers, neurites extended preferentially along the long axis. Yao et al. [54] combined electrospinning and molecular self-assembly technology to prepare an aligned fibrin hydrogel (AFG), which is a three-dimensional layered arrangement with a directional morphology to simulate the spinal cord tissue in the natural environment. The results proved that the motor function recovery rate of the AFG group was significantly faster than that of the control group and the stochastic fibrin hydrogel group (RFG). The AFG scaffold supplied an induction array for promoting directional host cell invasion, reconstruction of the vascular system, and axon regeneration, which could facilitate and buttress a wide range of axon regeneration and recovery of motor function. Zhang et al. [55] prepared aligned poly(3-hydroxybutyrate-co-3-hydroxyvalerate) PHBV nanofibers doped with polyethylene oxide (PEO) by electrospinning. Compared with randomly oriented nanofibers, aligned nanofibers showed clear guidance for cell extension. The surface of the nanotube enhanced the interaction between nerve cells and the scaffold. The aligned fiber was then treated with plasma and modified with laminin to fabricate a nerve conduit. The functionally arranged nanofibers effectively promoted the adhesion and proliferation of Schwann cells. In vivo animal experiments showed that the PHBV/PEO nanofiber nerve conduit coated with laminin on the inner surface could promote the regeneration of sciatic nerve defects. However, the defects of PHBV, including high crystallinity and large spherulite size caused brittleness and slow degradation (more than two years for complete degradation), extremely limiting its application in biomedicine [56].

Cell migration is critical to a wide range of pathological and physiological processes, including wound repair, metastasis, and embryogenesis [57–59]. The surface structure of biomaterials is essential to control their interaction with the biological environment [60]. It is well known that the micro- and nanoscale geometric cues presented by the extracellular matrix of cells affect cell behavior, such as migration, adhesion, and dispersion [61]. Therefore, it is important to understand the interaction between cells and different surface morphologies of artificial graft, which may improve the biological performance of medical implants [62]. Topological structure can also regulate cell functions, such as differentiation [63] or migration [64]. Researchers [65] studied the influence of surface topographical properties on the migration and dispersion of the cytoskeleton of stem cells. The surface of titanium alloy was firstly treated with a pulsed laser to form parallel grooves and matrix holes to evaluate the dispersion behavior of the cytoskeleton. The experimental results showed that the cell cytoskeleton was influenced not only by the surface roughness (Ra and Sa) but also by the surface flatness and area.

Microscale grooves could induce cells to form a high-aspect ratio cytoskeleton and restrict the migration and diffusion of the cytoskeleton. Li et al. [66] developed chitosan micropatterns with various pattern sizes for peripheral nerve regeneration. The 30 *μ*m size pattern showed the best regulation effect on Schwann cell migration. In addition, compared with the unpatterned sample, the micropatterned chitosan film could well retain the physiological function of SCs, indicating that chitosan micropatterning and SCs have good biocompatibility. Thus, chitosan micropatterning can effectively regulate the directional growth of SCs, and may have potential application value in the repair of peripheral nerve injury. Although chitosan has been widely used for constructing artificial grafts for tissue engineering, several disadvantages limit its application, such as its dependence on low pH and the risk of allergic reactions. The neonerve tissue migrated much faster in a chitosan conduit with surface micropatterning compared to the controlled conduit without topography [67]. Walter et al. [68] used a polystyrene colloidal template to fabricate a surface with controlled feature sizes. The results of fluorescence microscopy, WST analysis, and morphological analysis showed that the surface feature size had a strong effect on bone marrow-derived rat stromal cells (ST2). Quantitative analysis showed that compared with flat surfaces, when the surface feature size was reduced to less than 200 nm, cell adhesion, diffusion, viability, and activity were enhanced, while larger feature size was not conducive to cell adhesion. The effects of topography on nerve cell adhesion and migration are presented in Table 2.

### 2.3. Topography Affects Cell Alignment Growth and Neurite Guidance

The characteristic functions of complex tissues are largely determined by the shape and polarity of the cells [69–71]. By means of a process known as contact guidance, the polarity of many different cell types can be strongly influenced by substrate morphology [72–74]. Cells can adjust their arrangement according to the local geometry of their adhesion surface [75]. This process is called contact guidance and has been well established, but little is known about it. One of the challenges is the varied natural surface topologies associated with cell movement guidance. Many studies have reported that the surface topography could facilitate contact guidance of cell morphology and achieve controlled neurite outgrowth under the guidance of a combination of chemical cues and physical patterns, which was important in the treatment of damaged nerve in regenerative medicine [76, 77]. In a previous study, the effect of collagen gradient micropatterns on peripheral nerve regeneration was investigated. The prepared gradient collagen micropatterns could well regulate the directional growth of Schwann cells. At the same time, it could be used as gradient micropatterns with higher collagen concentration and larger pattern size [78].

Generally, at least two different types of stress fibers exist in the cell, one is the long top stress fiber arranged in the axial direction, and the other is the short base stress fiber arranged in the circumferential direction. A small guanosine triphosphatase (Rho) that regulates the assembly of stress fibers can realize the manipulation of stress fibers and achieve the purpose of guiding and controlling cell arrangement [79]. It has been documented that in organisms, cells treat the microscopic nanofiber matrix network as a physical signal, which affects the arrangement of cells in the range of tens to hundreds of microns. Many studies reported that the microgroove could cause neurite arrangement, and the surface topography increases the stimulation of discrete retinal neurites and enhances the orientation of neurites [80–83]. Observations have also shown that topographical features could provide guidance for the direction and growth of hippocampal neurons, and these directional cues contribute to the long-term organization of axons and dendrites [82]. In addition, it has also been reported that the growth cone of neurons can perceive nanoshaped extracellular matrix tips to produce stable neurites [83]. Moreover, it was found that microRNA is involved in the growth of neurites guided by topological cues [84]. The engineered mussel protein glue-based nanofiber catheters were also used to carry out multidimensional biologically inspired strategies to accelerate the regeneration of functional nerves [33]. The results showed that the AFG biomimetic composition is comprised of a protein of a natural fiber cable, and the aligned organization could facilitate rapid biological function recovery in peripheral nerve regeneration [85].

According to previous studies, the scale of the topography has an effect on the growth of neurites on electrospun nanofibers. In previous studies, it was found that the fiber diameter strongly affects the morphology and differentiation [86], neurite length [87], and neurite arrangement of nerve cells [88–90]. Topographical structures, such as the width and height of a groove have a significant impact on the alignment and elongation of various types of cells. The aligned PLLA nanofiber scaffold highly improved the neurite outgrowth, NSC displayed an obvious bipolar elongated morphology of the cell soma, and the neurites grew outward [86]. Topographical size was also shown to have an important influence on the differentiation and growth of nerve cells. The results of the research may provide valuable reference information for developing effective tissue remodeling matrices and optimizing existing biomaterials for neural tissue engineering applications. The neatly arranged electrospun fibers have shown great promise in promoting the growth of directed neurites in cells and animal models. Although electrospun fiber diameter affected cell behavior, it is unclear how directional spun fiber scaffolds of different diameters affect neurite growth and Schwann cell migration. Therefore, the study firstly established highly arranged electrospun fiber scaffolds with different diameters, and then evaluated the neurites and stem cell behavior of dorsal root ganglion (DRG) explants. Three groups of highly aligned electrospun polylactic acid (PLLA) fibers (1325 + 383 nanometers: large diameter fibers; 759 + 179 nanometers: medium diameter fibers; and 293 + 65 nanometers: small diameter fibers) were produced. Moreover, the extension of DRG neurites in chicks was found to be affected by the fiber diameter [88]. Small fiber diameters (293 ± 65 nm) did not promote the extension of DRG neurites, moderate fiber diameters (759 ± 179 nm) could promote the directional expansion of neurites, while large fiber diameters (1325 ± 383 nm) could obviously promote the directional growth and extension of DRG neurites. The packing density of fibers may also affect the extension of axons and the migration of Schwann cells [88]. Therefore, when constructing aligned electrospun fiber constructs for nerve regeneration applications, careful consideration should be given to fiber diameter and spacing between fibers. As a material with a relatively slow degradation rate, PLLA may cause some problems, such as incomplete degradation and lower local pH. Therefore, PLLA implants may cause late inflammation, cell necrosis, and delayed axonal recovery in the body [91], which should also be considered for future clinical applications.

Human embryonic stem cell-derived neural stem cells on silk fibroin scaffolds with different diameters (i.e., 400 and 800 nanometers) and orientations (i.e., random and arrangement) were used to analyze the effects of fiber diameter and arrangement on nerves derived from human embryonic stem cells including cell viability, neuron differentiation, and neurite growth. Compared with random thyroid-stimulating hormone-releasing factor scaffolds, the arranged thyroid-stimulating hormone-releasing factor scaffolds could significantly promote the differentiation and axon growth of human embryonic stem cell-derived neurons. In addition, on the aligned 400-nanometer fibers, cell viability, neuron differentiation, and neurite outgrowth were greater than those on the aligned 800-nanometer fibers. Their results indicated that the 400-nanometer directional thyroid-stimulating hormone-releasing factor scaffold was more suitable for the development of neural stem cells derived from human embryonic stem cells, which was helpful for optimizing the therapeutic potential of human embryonic stem cells for nerve regeneration [83]. However, although natural silk has good mechanical properties, the mechanical properties of silk protein after degumming are impaired, which further affects the performance of silk fibroin nerve conduits. A previous study showed that the aligned PLLA fiber scaffold could promote the elongation of neural stem cells, and their neurites grew along with the fiber direction, while the fiber diameter had no significant effect on cell orientation. Interestingly, it was reported that the NSC differentiation rate on nanofibers was higher than microfibers, but it had no relationship with fiber orientation degree [86]. Besides, the aligned silk fibroin nanofibers with different diameters were shown to support astrocyte attachment and growth. An obvious enhancement of the astrocyte migration area was observed on SF scaffolds with a diameter of 400 nm compared to those with a diameter of 1200 nm [89]. In our previous research, we also used a combination of micromodeling and freeze-drying to prepare porous chitosan micropatterns with different sizes of surface ridges/grooves. The Schwann cells on the chitosan micropattern appeared to display orientation adhesion and began to grow in a certain direction after having been cultured for 2 h. After further culture for 3 and 5 d, the minimum orientation angle and maximum length appeared on the 30/30 *μ*m micropattern. Therefore, the results showed that the porous chitosan micropatterns with a suitable size could well regulate the growth of Schwann cells and had potential application value in nerve regeneration [67].

The rapid vascularization of the nerve graft at the wound site is very important for nerve regeneration [92]. Vascular endothelial cells (VEC) are one of the most important core cells for angiogenesis [93]. Endothelial cells change their phenotype and could grow guided by the topography. The topography with a larger microsize (width range of 4.8 to 9.9 *μ*m and height range of 1015 to 2169 nm) resulted in cell alignment and inhibited vascular network formation, while the topography with a nano/microsize (width range from 1.5 *μ*m to 3.8 *μ*m and height range from 176 nm to 780 nm) could lead to the formation of early vascular networks [94]. Moreover, it was found that the scaffolds with aligned topologies could form a fused endothelium with enhanced endothelial cell (EC) adhesion for vascularization. The cells on the scaffolds with aligned fibers of 100 nm and 300 nm diameter had a significantly larger aspect ratio and long axis length compared with those on aligned fibers of 1200 nm and random fibers. Fiber orientation had a great influence on the alignment of guided cells on the scaffolds with diameters of 100 nm and 300 nm, while the cells on the 1200 nm scaffold did not show aligned growth [95]. Previous reports have demonstrated that cell-topography interactions can affect the gene expression, neurite branching, growth, and reprogramming efficiency of induced neurons (iNs) [96]. Cell arrangement and neuron guidance stimulated by topological structure with different sizes is summarized in Table 3.

### 2.4. Topography-Nerve Cell-Relevant Mechanism

**Figure 2:**
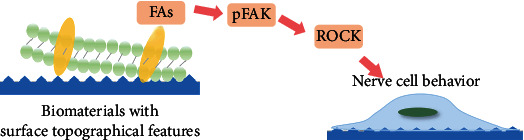
Schematic diagram of material surface topographical features regulating nerve cell behavior. Nerve cells perceive stimuli of different topographical features through the focal adhesion-pFAK-Rho A signaling pathway.

Cell responses to topographical cues can be caused by direct changes in cytoskeletons and indirect changes in signaling pathways. However, the mechanism of how neuronal cells sense and react to topography is rarely reported. For nonneuronal cells, it is generally believed that the recognition mechanism between cells and topographical features in the extracellular environment is based on integrin adhesion molecules [97]. Integrin expresses widely in the nervous system. The presence of integrin is related to various developmental processes of the nervous system, such as neuron migration, axon growth, and guidance through attachment to extracellular matrix proteins such as tenascin laminin and fibronectin [98–100]. In addition, it has been detected in many growth cones of focal adhesion protein, such as tallin and vinculin, indicating that the integrin-cytoskeleton coupling may play a major role in the movement of growth cones and the processes of neurons [100, 101]. Therefore, most of the reported influence mechanisms of topographical features on nerve cells mainly focused on the activation of integrin, the cytoskeleton, ROCK signaling, and focal adhesion [102–104]. Activation of integrin leads to the organization of focal adhesions (FAs), the actin filaments that connect integrin to the cytoskeleton [105]. FAs include proteins such as talin or vincinin, which transfer mechanical forces through the whole actin-myosin cytoskeletal network, and directly leads to changes in cytoskeletal assembly, and finally cell morphology [97, 106]. In addition, the FA proteins are comprised of an activation signal cascade of biochemical signals such as paxillin [106]. These cascades, which include activation of phosphorylation-mediated signaling pathways and activation of G-protein- (such as Rho-) mediated signaling pathways (such as ROCK), may cause long-term changes in cell proliferation, differentiation, survival, and transcriptional regulation [97]. A receptor for activated protein kinase C (RACK1) inhibits the nanoscale response to contact guidance and promotes adhesion as well [107]. Under the different characteristics of the surface topographical structure, the single adhesion may change to the protein aggregation and reorganization, which affects the quantity and distribution of FAs [108]. In short, the surface topographical cues of the material can regulate the behavior of nerve cells through the focal adhesion-pFAK-RhoA signaling pathway (Figure 2).

## 3. Outlook

Tissue engineering is an interdisciplinary research field that applies materials and biological sciences to create artificial organs and tissue matrices [13]. Micro-/nanostructured biomaterials with specific characteristics and functions are in great demand in biomedical applications. Neural tissue engineering relies on combining biological materials and external cues, such as cell-matrix interactions [109]. For application in peripheral nerve regeneration, the surface topography of the biomaterial plays an important role in regulating nerve cell behavior. The surface topography could present with different morphologies and structures, thereby providing a superior microenvironment that is suitable for nerve tissue regeneration. Many researches have reported that the cell proliferation, differentiation, adhesion, migration, arrangement, and neurite guidance could be manipulated by the interaction of cell-biomaterial surfaces with different topological structures and sizes. In addition, the unique topography and structure of the surface of biomaterials may affect the differentiation type of nerve cells. Further on, the surface topographical specification of biomaterials (surface roughness, surface groove size and direction, and surface pore size and distribution) will significantly affect the molecular pathways that control the fate of nerve cells.

## 4. Conclusions

This review gives a broad overview of the powerful influence of the topological structure of the material surface on different neuronal cell types, including neurons and glial cells. Feature roughness, groove size and orientation, and surface pore size are important parameters that regulate cell behavior on topological structures. The review illustrated that different topographical features have a great impact on cell proliferation, differentiation, adhesion, migration, arrangement, and neurite guidance. In particular, the cell's behaviour would be most significantly improved across different topographical features. Future research will further reveal the regulatory mechanisms of topography on cellular response, which will significantly influence nerve regeneration. The correct design and fabrication of appropriate dimensions and shapes of topographical features will be helpful for the development of artificial nerve grafts with excellent biological performance in nerve tissue engineering and neuroregenerative medicine.

## Figures and Tables

**Table 1:** Summary of the influence of topographical features on the proliferation and differentiation of cells.

Feature type	Cell type	Effects on cell behaviors	Reference
PLA nanofiber stents	Neurons and glial cells	Promote neuron and glial cell growth	[32]
Mussel protein glue-based nanofiber conduit	Nerve and Schwann cells	Promote nerve and Schwann cell proliferation and differentiation	[33]
Nanotextured glass coverslips	PC12 cell	Promote the proliferation of neuronal cells and provide treatment for central nervous system trauma	[35]
A customizable multilevel architecture array	Neurons	Enhance the differentiation of neurons	[36]
The engineered concave microarray	Cardiomyocytes and neurons	The differentiation of cardiomyocytes and neurons	[37]
Polycaprolactone/polyethylene oxide–polycaprolactone-blended fiber materials using electrospinning technology	Neural stem cells	Promotes the growth of rat neural stem cells, differentiates into neurons and astrocytes	[38]

**Table 2:** Summary of the effects of sizes on cell adhesion and migration.

Feature type	Effects on cell behaviors	Reference
Prepared poly(L-lactic acid-co-e-caprolactone) (P(LLA-CL)) nanofibers	Have different regulatory effects on macrophage activation	[49]
Polylactide-glycolide copolymers were used to prepare random and longitudinally arranged electrospun fibers such as two-dimensional films and three-dimensional films	Increase in the adhesion of Schwann cells	[50]
Arranged nanofibers	Neurites extended preferentially along the long axis	[53]
Poly(3-hydroxybutyrate-co-3-hydroxyvalerate) PHBV nanofibers doped with polyethylene oxide (PEO)	Promoted adhesion and proliferation of Schwann cells	[55]
Micron-scale groove	Limiting the migration and diffusion of the cytoskeleton	[65]
Used a polystyrene colloidal template to make a surface with controlled feature sizes	When the surface feature size is reduced to less than 200 nm, cell adhesion, diffusion, viability, and activity are enhanced	[68]

**Table 3:** Summary of the effects of sizes on cell alignment and neurite guidance.

	Diameter	Material	Cell type	Effects on cell behaviors	Reference
Silk nanofibers/scaffold	400 ± 67 nm 800 ± 35 nm	Silk fibroin	Human embryonic stem cell- (hESC-) derived neural precursors (NPs)	Cell viability, neuronal differentiation, and neurite outgrowth are greater on aligned 400 nm fibers	[83]
Poly (L-lactic acid) (PLLA) nano/microfibrous scaffolds	300 nm (nanometer scale) 1.5 mm (submicron scale)	PLLA	Neural stem cells (NSCs)	Aligned nanofibers highly supported the NSC culture and improved the neurite outgrowth	[86]
Silk fibroin (SF)	400 nm 1200 nm	Silk fibroin (SF)	Astrocytes	Significant increase in astrocyte diffusion area on SF scaffolds at 400 nm	[89]
Poly-L-lactic acid (PLLA) fibers	293 ± 65 nm 759 ± 179 nm 1325 ± 383 nm	Poly-L-lactic acid (PLLA) fibers	DRG neurites in chicks	Large fiber diameters (1325 ± 383 nm) obviously promote the directional growth and extension of neurites	[88]
Gradients	Wavelengths ranging from 1.5 *μ*m to 3.8 *μ*m and amplitudes ranging from 176 nm to 780 nm	Polydimethylsiloxane (PDMS)	Human pulmonary microvascular endothelial cells (ECs)	Caused formation of early networks that can be stabilized by the use of the guidance layer of adipogenic stromal cells (ASC)	[94]
	Wavelengths ranging from 4.8 to 9.9 *μ*m and amplitudes ranging from 1015 to 2169 nm			Caused cell alignment and inhibited vascular endothelial network formation	
Electrospun scaffolds	100 nm and 300 nm 1200 nm	Poly(*ε*-caprolactone) (PCL) and type I collagen	Endothelial cell (EC)	Significantly larger aspect ratio and long axis length	[95]

## Data Availability

Data sharing is not applicable to this article as no datasets were generated or analyzed during the current study.
